# The HMA-domain protein Ict1 is required for Ferroptosis in the rice blast fungus

**DOI:** 10.3389/fpls.2025.1576086

**Published:** 2025-06-05

**Authors:** Qing Shen, Madiha Natchi Samu Shihabdeen, Ken Harata, Naweed I. Naqvi

**Affiliations:** ^1^ Temasek Life Sciences Laboratory, 1 Research Link, Singapore, Singapore; ^2^ Department of Plant Life Science, Ryukoku University, Seta, Shiga, Japan; ^3^ Department of Biological Sciences , National University of Singapore, Singapore, Singapore

**Keywords:** cell death, copper, Ferroptosis, iron, lipid peroxidation, mitochondria, pathogenesis, rice blast

## Abstract

Lipid peroxidation and iron-dependent cell death, Ferroptosis, in conidia plays a crucial role in ensuring proper infection structure formation and function in the rice blast fungus *Magnaporthe oryzae*. However, the distribution of such important cation(s) in regulating precise developmental cell death remains unexplored. Here, we characterized the role of an iron-copper chaperone (Ict1) and a copper transporter (Ccc2) in growth and pathogenesis in *Magnaporthe*. These Heavy-Metal-Associated domain containing proteins, particularly Ict1, were found to be important for Ferroptosis in rice blast conidia. Loss of Ict1 or Ccc2 induced significant viability in the three conidial cells in contrast to the sequential demise therein in the wild-type *M. oryzae.* Furthermore, an increased accumulation of oxidized lipids at the plasma membrane was evident in wild-type, but not in the *ict1*Δ or *ccc2*δ conidial cells undergoing pathogenic differentiation. The *ict1*δ showed a lack of iron accumulation in conidia at the crucial time points, and such defects in cation homeostasis and cell death were suppressed significantly upon exogenous provision of ferric ions, and to a minor extent with copper. Interestingly, the *ict1*δ conidia exhibited diminished mitophagy and in turn led to a profound increase in mitochondrial membrane potential and stability suggestive of enhanced organellar function that correlates negatively with conidial cell death via Ferroptosis. Lastly, Ict1-GFP was found to be predominantly cytosolic and excluded from the vacuoles during the vegetative and infection-related development in rice blast.

## 1 Introduction

Ferroptosis is a highly conserved, iron-dependent cell death mechanism triggered by peroxidation of membrane lipids to lethal levels (Dixon et al., 2012; Stockwell, 2022). Such iron enabled cell demise is also conserved in the fungal pathogen, *Magnaporthe oryzae*, that causes rice blast disease, and plays an essential role in its pathogenic development (Shen et al., 2020). To invade rice plants, asexual spores or conidia of *M. oryzae* need to germinate and form the infection structure, which is a new cell-type named appressorium, at the tip of the germ tube (Eseola et al., 2021). Mature appressorium with a proper melanin layer underneath its cell wall is able to accumulate high turgor pressure to physically penetrate rice leaves though a narrow, rigid penetration peg, and thus initiates *in planta* invasive growth in host cells (Talbot, 2019; Eseola et al., 2021). The conidium of *M. oryzae* contains three cells, which sequentially initiate Ferroptosis one after another when the appressorium at the other end of the germ tube undergoes maturation (Shen et al., 2020). Disruption of such iron-dependent cell death inevitably leads to defects in rice infection (Shen et al., 2020), highlighting Ferroptosis in the conidium as an essential part of the pathogenic development of the rice blast fungus. Such compartmentalized Ferroptosis is regulated by NADPH oxidases which serve as a source of reactive oxygen species (ROS) for lipid peroxidation in the membranes (Shen et al., 2020). The lysosome/vacuole-based cellular degradation and recycling mechanism, autophagy, is also tightly connected with Ferroptosis, since the autophagy defective mutant in *M. oryzae*, *atg8*Δ (Deng et al., 2009), suffers severe iron and redox deficiency and fails to undergo Ferroptosis (Shen et al., 2020). In addition to the non-selective autophagy, the *atg24*Δ mutant is defective in mitophagy and fails to target dysfunctional or excess mitochondria for vacuolar degradation (He et al., 2013), demonstrates similar iron deficiency and Ferroptosis defects (Shen et al., 2024), thus further linking iron-dependent Ferroptosis with mitochondrial activities and homeostasis. How is Ferroptosis tightly controlled through iron regulation in the rice blast fungus, however, remains elusive.

Iron uptake in budding yeast, *Saccharomyces cerevisiae*, is well-characterized and is known to been closely connected to copper homeostasis, because iron uptake by the transmembrane permease Fe TRansporter 1 (Ftr1) requires it to be oxidized from ferrous to ferric ion by the multicopper oxidase FErrous Transport 3 (Fet3) (Askwith et al., 1994; De Silva et al., 1995; Stearman et al., 1996; Hassett et al., 1998; Wang et al., 2003). A conserved copper relay system involving the cytosolic copper chaperone AnTioXidant 1 (Atx1) and the secretory vesicle copper transporter Cross-Complements Ca^2+^ phenotype of *csg1*Δ 2 (Ccc2), delivers copper from the cell-surface Copper TRansporter 1 (Ctr1) to insert into Fet3 (Yuan et al., 1995; Lin et al., 1997; Pufahl et al., 1997; Yuan et al., 1997; Payne and Gitlin, 1998; Portnoy et al., 1999; Huffman and O’halloran, 2000 ;Banci et al., 2001; Xiao et al., 2004). Not surprisingly, *atx1*δ and *ccc2*δ deletion mutants are deficient in iron acquisition, and such defects can be reduced/suppressed by copper treatment (Yuan et al., 1995; Lin et al., 1997). Such pathway connecting iron homeostasis with copper availability is tightly controlled by a set of transcription factors including Activator of Ferrous Transport/Activator of Fe Transcription (Aft) 1 and Aft2 as well as Yeast AP-1 (Yap) 5, which individually or collaboratively regulate the expression of above-mentioned genes (Yamaguchi-Iwai et al., 1996; Lin et al., 1997; Macisaac et al., 2006; Hu et al., 2007; Venters et al., 2011; Pimentel et al., 2012). It is noteworthy that activity and/or subcellular localization of the iron-sensing Aft1, Aft2 and Yap5 is determined by the iron-sulfur cluster sourced from mitochondria as a cofactor (Gupta and Outten, 2020). Unfortunately, none of these transcription factors is conserved in *M. oryzae*, implying an evolutionary divergency between the budding yeast and the rice blast fungus. Furthermore, the rice blast fungus likely relies mainly on internal nutrient sources for pathogenic development before host penetration since environmental resources except for water are extremely limited or restricted. Surprisingly, part of the copper-dependent iron acquisition pathway, including Atx1and Ccc2, is conserved in *M. oryzae*, implying a modified usage of such functions for iron release from different intracellular sources. In addition to iron, overload of copper has also been reported to induce death in cultured mammalian cells by disrupting the function of tricarboxylic acid cycle in mitochondria (Tsvetkov et al., 2022). It will be interesting to know whether such copper toxicity and Ferroptosis are interconnected or not in the blast fungus.

Atx1 and Ccc2 orthologs in *Colletotrichum orbiculare* have been characterized and CoAtx1 was renamed as Iron Copper Transporter 1 (Ict1) (Harata et al., 2020). Loss of Ict1 function leads to insensitivity to the fungicide ferimzone and a defect in melanin synthesis (Harata et al., 2020). Not surprisingly, such defects can be completely suppressed by adding copper to the medium or directly to the fungus (Harata et al., 2020), thus supporting the conserved role of Ict1 as a copper chaperone in *C. orbiculare.* Atx1/Ict1 and Ccc2 orthologs in *M. oryzae* have also been characterized for their role in ferimzone resistance and melanin biosynthesis (Harata et al., 2020). However, their role in iron and/or copper homeostasis and in Ferroptosis regulation *per se* remains unclear. Here, we demonstrate that the cytosolic HMA-domain containing protein, Ict1, indeed regulates Ferroptosis through iron and redox homeostasis; and an active involvement of mitochondrial function in cation regulation is evident in the blast fungus. Overall, a new iron-homeostasis-based Ferroptosis regulator is identified, which could be a potential link in vegetative-to-pathogenic transition and could serve as a fungicide target for controlling the devastating blast disease in rice.

## 2 Materials and methods

### 2.1 Fungal strains

*Magnaporthe oryzae* wild-type strain P2, and the isogenic deletion mutants *ict1*Δ (Harata et al., 2020) and *ccc2*Δ (Harata et al., 2020) were obtained from Ken Harata’s group in Ryukoku University, Japan. The in-locus tagging strain expressing *ICT1-GFP* was made through the Agrobacterium T-DNA mediated transformation (ATMT) based homologous recombination method using *M. oryzae* strain B157 from the Directorate of Rice Research (Hyderabad, India) as the parent strain for fungal transformation.

The vector used for such homologous recombination was constructed using the ClonExpress MultiS One Step Cloning Kit (Vazyme, C113). Briefly, the 1170 bp genomic sequence before *ICT1* stop codon (5’ arm), the eGFP encoding sequence (without start codon) together with the Bar resistant cassette, and the 1009 bp genomic sequence include the *ICT1* stop codon and 3’UTR (3’ arm) were PCR amplified, purified, and then assembled with a linearized empty vector pFGL815 (Addgene #52322, Naweed Naqvi’s lab) using Vazyme kit. Once sequence of such vector was verified, it was electroporated into the Agrobacterium AGL1, which was subsequently used to transform B157 through Agrobacterium T-DNA mediated transformation (Rho et al., 2001). Positive transformants were selected on basal medium (1.6 g/L yeast N_2_ base without amino acids or ammonium sulphate, 2 g/L Asparagine, 1 g/L NH_4_NO_3_, 10 g/L glucose, 2% agar, pH was adjusted to 6 using 1 M Na_2_HPO_4_) supplied with 50 mg/L Basta/Glufosinate ammonium (Sigma-Aldrich, 32874). Correct in-frame insertion of GFP before the stop codon at the *ICT1* locus was verified in individual transformants by requisite PCR and nucleotide sequencing using the indicated primers (**Supplementary Figure S1A, B**). Ict1-GFP expression and localization was further confirmed by confocal microscopy. Details of primers for vector construction and strain verification are provided in **Supplementary Table S1**.

### 2.2 Fungal culture conditions

For selecting fungal transformants, basal medium plates were incubated at 28°C in darkness for vegetative growth until Basta resistance was obvious.

For conidiation, *M. oryzae* strains were grown on prune agar plates (1 g/L yeast extract, 2.5 g/L lactose, 2.5 g/L sucrose, 4% (v/v) Del Monte prune juice, 2% agar, pH was adjusted to 6.5 using 10 M NaOH) and kept at 28°C in darkness for two days and then exposed to continuous light at room temperature for another five to seven days.

### 2.3 Cell death quantification

Fresh conidia were harvested from *M. oryzae* strains grown on prune agar medium, and 30 μL water droplets containing 2×10^5^ conidia per mL in the presence or absence of 10 µM FeCl_3_ or CuSO_4_ were then inoculated on hydrophobic cover glasses (Matsunami, Japan) to induce *M. oryzae* pathogenic development. Conidia capable of forming a mature or immature appressorium were used for cell death quantification at 24 hours post inoculation (hpi) using 1% trypan blue staining and/or requisite epifluorescent nucleus-tagged (3xNLS-mCherry) strains of *M. oryzae* (**Supplementary Figure S1C, D**). Nuclear degeneration as judged by mCherry signal being vacuolar or completely degraded therein was used as a proxy for cell death. Time lapse confocal microscopy was used to study the temporal dynamics of cell death in conidia in such nucleus-tagged strains. A conidium was considered dead when all three cells within it were inviable. Otherwise, it was regarded as viable/alive. The abnormal cases, albeit minor, that both conidium and appressorium were dead was not included in the “dead” category. Student’s *t*-test was used to distinguish significant differences between wild type and mutants with or without cation treatment.

### 2.4 Confocal microscopy

Mycelia undergoing vegetative growth were prepared by cutting a very thin piece from the basal medium, while pathogenic development of *M. oryzae* was induced by inoculating conidia on Matsunami cover glasses. Conidia at indicated time points were then used for fluorescence imaging directly or stained with 10 µM BODIPY™ 581/591 C11 (D3861; Thermo Fisher), 1 µM Calcein-AM (C3099; Invitrogen) or 250 nM Tetramethylrhodamine ethyl ester perchlorate (TMRE, Sigma, 87917) for 15-30 min at room temperature.

Fluorescence imaging was performed using the laser scanning confocal microscope system TCS SP8 X (Leica Microsystems), controlled by the Application Suite X software package (Leica Microsystems, version 3.5.7.23225). Particularly, the HC Plan Apochromat CS2 63×/1.40 oil immersion objective and the white light laser controlled by the AOTF (Acousto-Optical-Tunable-filter) were used for Calcein-AM (excitation, 494 nm; emission, 510-550 nm), GFP (excitation, 488 nm; emission, 500-550 nm), and TMRE (excitation, 540 nm; emission, 580-610nm) imaging. The Matsunami micro slide glass (S7213; Matsunami, Japan) and the HCX Plan Apochromat lambda blue 63×/1.20 water immersion objective were used for BODIPY™ 581/591 C11, with the Argon laser (excitation, 488 nm; emission, 500-535 nm) for exciting the oxidized form of such fluorescent probe, while the white light laser (excitation, 561 nm; emission, 573-613 nm) was used for the non-oxidized variant. Fluorescence signals at Z stacks of 10 to 15 sections (0.5 or 0.7µm-spaced) were detected using the Leica Hybrid Detector. For live cell imaging, freshly harvested conidia were inoculated on glass bottom culture dishes (P35G-0-14-C; MatTek Corp., Ashland, MA, USA) for the indicated time period. Epifluorescence of Histone H1-GFP or 3xNLS-mCherry was captured using the Leica TCS SP8 X inverted microscope system (Leica Microsystems) with the aforementioned settings and a white light laser for Histone H1- GFP (excitation, 488 nm; emission, 500–550 nm) and mCherry (excitation, 543 nm; emission, 610– 680 nm).

## 3 Results

### 3.1 Ict1 and Ccc2 modulate iron-dependent cell death in *M. oryzae* conidia

**Figure 1:**
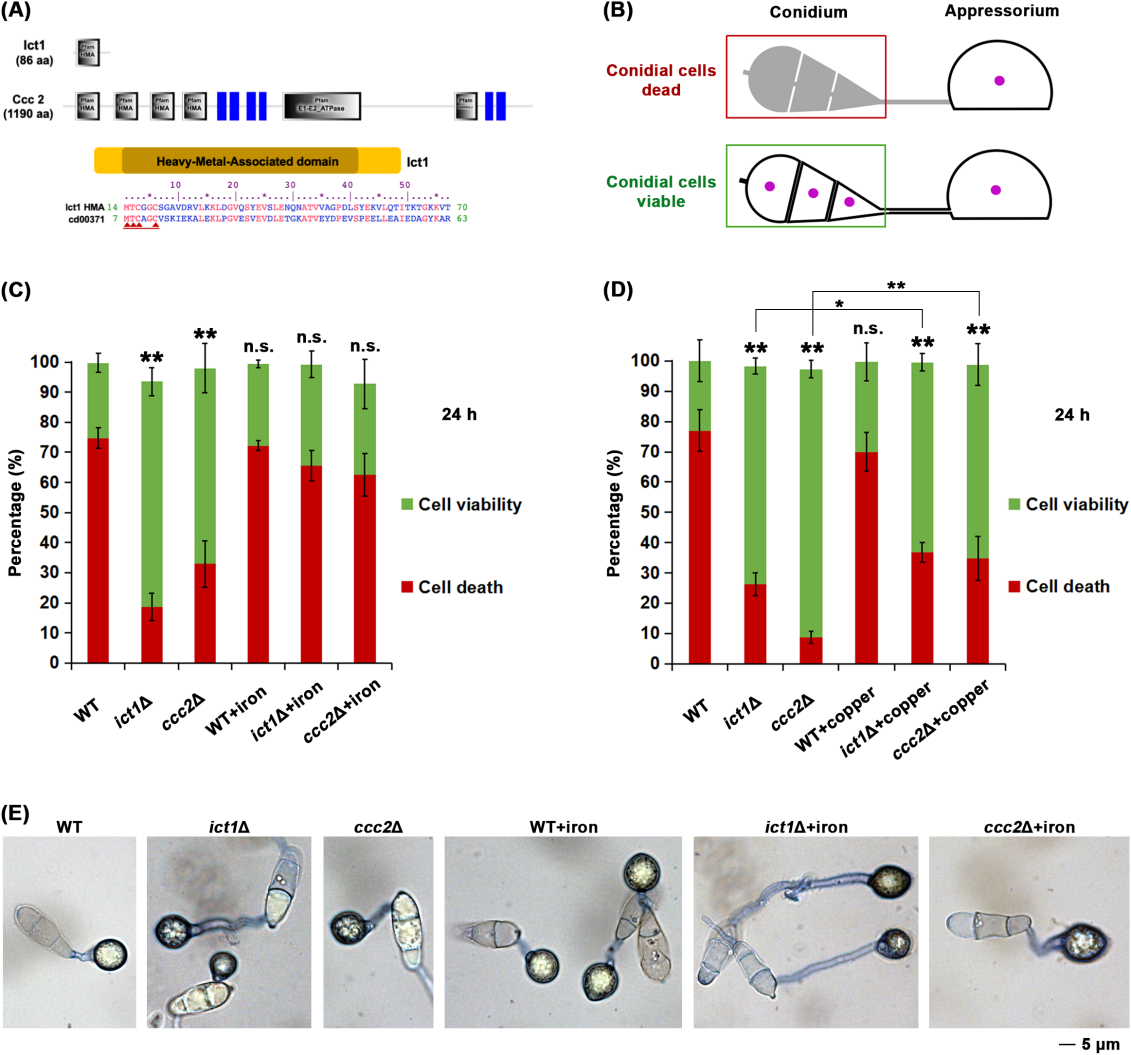
Ict1 and Ccc2 are required for the iron-mediated developmental cell death in the rice blast fungus *M. oryzae.* **(A)** Schematic representation of the domain organization of the iron-copper chaperone and transporter, Ict1 and Ccc2, respectively, in *Magnaporthe*. Ict1 possesses a single HMA domain, whereas Ccc2 shows a typical P-type ATPase motif along with 4 copies of HMA domain, the consensus sequence thereof and its similarity to the PFAM cd00371 are depicted. Red arrows indicate the cation (copper/iron) binding sites. **(B)** Diagrammatic presentation of the conidial cell death that occurs in the wild-type *M. oryzae* during the infection-related morphogenesis i.e. appressorium formation. The 3 conidial cells and the infection structure (appressorium, which remains viable and functional) are shown. Perturbation of Ferroptosis/cell death renders conidial cells viable. Same color coding for viability and death is retained in the next panel. **(C)** Loss of Ict1 or Ccc2 leads to marked reduction in Ferroptotic cell death, which can be significantly restored by exogenous iron in the rice blast fungus. Conidial cell viability (green) or death (red) in the wild type (WT), *ict1*Δ or *ccc2*Δ was quantified at 24 hpi in the presence or absence of the indicated amounts of iron/ferric ions. Data presented as mean ± SD (3 technical replicates, n=100 conidia for each time point per strain per replicate). ** (p < 0.01) and * (p < 0.05) indicate significant differences, while n.s. refers to no significant difference detected in comparison to the WT at the corresponding time points. Experiment has been repeated thrice. **(D)** Exogenous copper restores cell death to some extent in the *ict1*Δ or *ccc2*Δ conidia. Conidial cell viability (green) or death (red) in the wild type (WT), *ict1*Δ or *ccc2*Δ was quantified at 24h in the presence or absence of copper ions. Data presented as mean ± SD (3 technical replicates, n=100 conidia for each time point per strain per replicate). ** (p < 0.01) and * (p < 0.05) indicate significant differences, while n.s. refers to no significant difference detected in comparison to the WT at the corresponding time points. Experiment has been repeated thrice. **(E)** Representative images of conidia of the indicated genotypes stained with trypan blue and corresponding to the quantification of Ferroptotic death or viability in conidial cells shown in panel **(C)** above. Please refer to the Methods section and **Supplementary Figure 2B, C** for relevant details about detailed cell death quantification. Exogenous iron supplementation significantly restores Ferroptotic cell death in *ict1*Δ or *ccc2*Δ conidia. Conidial cell viability or death in the wild type (WT), *ict1*Δ or *ccc2*Δ was quantified at 24 hpi in the presence or absence of iron/ferric ions. Scale bar equals 5 microns.

Ict1 contains a Heavy-Metal-Associated (HMA) domain (**Figure 1A**) and is predicted to be a copper chaperone in *M. oryzae.* To assess the role of Ict1 in cellular iron homeostasis and Ferroptosis regulation in *M. oryzae*, conidial cell death (**Figure 1B**, **Supplementary Figure S2A-C**) was quantified in detail in the wild type, *ict1*Δ and *ccc2*Δ mutant. Majority of the wild-type conidia completed such conidial cell death at 24 hpi, whereas *ict1*Δ or *ccc2*Δ conidia, unlike their wild-type counterparts, remained mostly viable (**Figure 1C, D**; **Supplementary Figure S2B, C**). Such cell death defect was rescued to the wild-type level upon iron treatment (**Figure 1C**; representative images shown in **Figure 1E**), but not with exogenous copper supplementation (**Figures 1D**; **Supplementary Figure S2B, C**), indicating that a shortage of available iron in the conidia causes such cell death defects. A time course analysis using live cell imaging of nucleus-tagged strains further confirmed the temporal dynamics of cell death and lack thereof in the wild-type and the *ict1*Δ mutant conidia, respectively (**Supplementary Figure S3**).

In yeast, Atx1/Ict1 transfers copper to the Cu^+2^-transporter, Ccc2, for final insertion into the multicopper oxidase Fet3 responsible for iron uptake. Ccc2 in *M. oryzae*, in contrast to Ict1, contains several HMA motifs, and a P-type ATPase domain (**Figure 1A**). Therefore, we also included *ccc2*Δ mutant in our study to assess its response to iron or copper treatment. Similarly, majority of *ccc2*Δ conidia were incapable of undergoing conidial cell death, and such defect was suppressed by iron supplementation but, rather surprisingly, not with exogenous copper (**Figures 1C, D**). However, exogenous copper restored cell death only marginally or minimally (compared to iron) in the *ict1*Δ and *ccc2*Δ conidia (**Figure 1D**; **Supplementary Figure 2**), which is consistent with the roles of Atx1/Ict1 and Ccc2 in copper chaperone and/or iron transport functions in yeast. Together, we infer that Ict1 and Ccc2 are capable of mediating iron availability and are key regulators of the associated developmental cell death/Ferroptosis in conidia during infection-related morphogenesis in *M. oryzae*.

### 3.2 Ict1 mediates Ferroptosis through cellular iron and redox homeostasis

**Figure 2:**
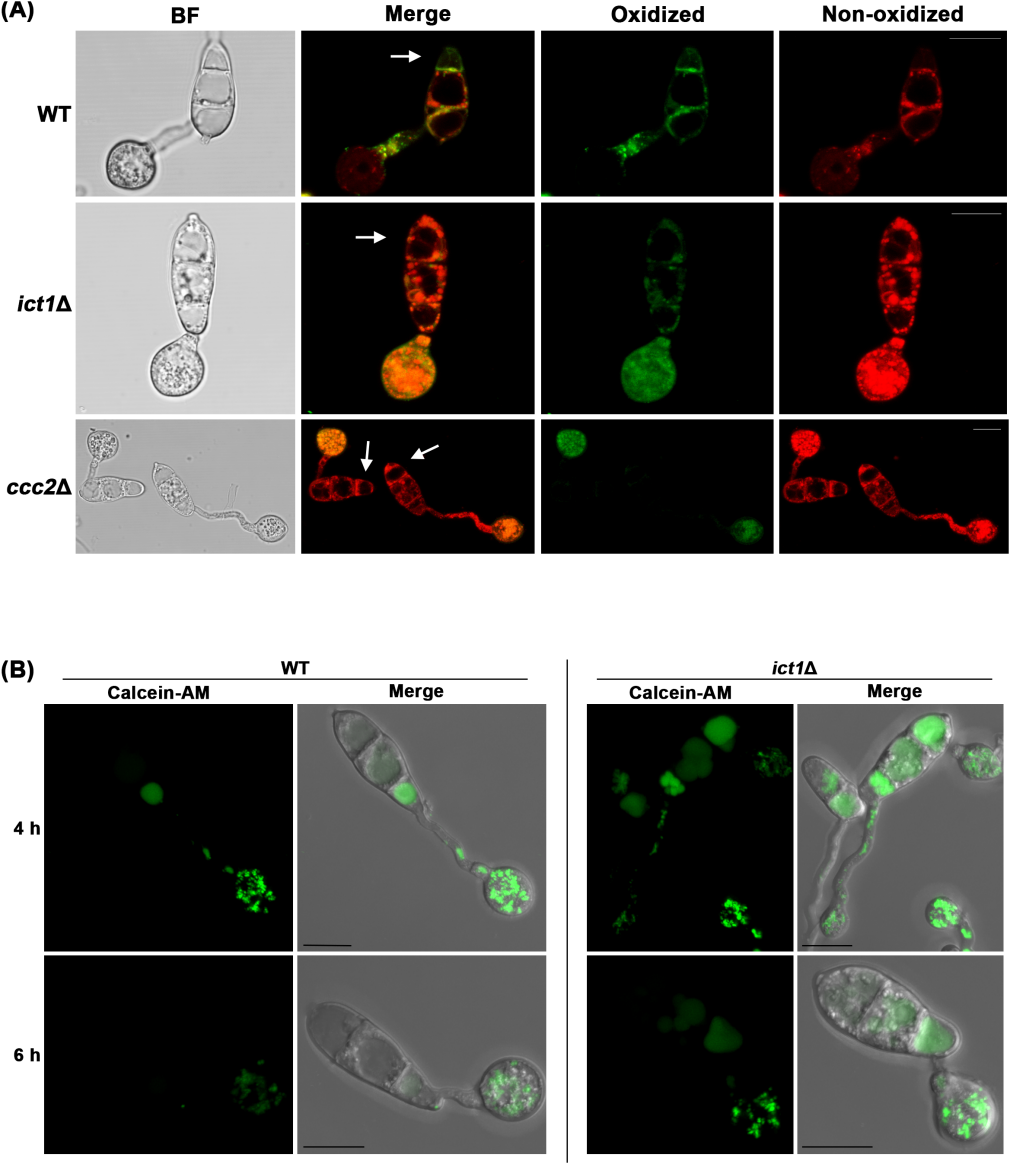
Loss of Ict1 function leads to significant reduction in lipid peroxidation and iron deficiency in *M. oryzae.* Confocal micrographs depicting lipid peroxidation at 7 hpi using BODIPY™ 581/591 C11 **(A)** or detecting iron deficiency in *ict1*Δ conidia, germ tubes and appressoria as assessed by staining with the FRET sensor Calcein-AM at 4 and 6 hpi **(B)**. Merged refers to combination of green epifluorescences with red ones or the bright field images. Arrow heads mark the dying terminal conidial cells. Bars = 10 µm.

To validate that *ict1*Δ and *ccc2*Δ are defective in undergoing Ferroptosis, lipid peroxidation in wild-type and such mutant conidia was assessed through ratio-metric analysis of BODIPY™ 581/591 C11 undecanoic acid, which is localized in membrane and shows a shift in fluorescence emission from red to green upon oxidation. Indeed, intense green signal indicative of oxidized lipids was observed in the plasma membrane of the terminal conidial cell in the wild type during initiation of Ferroptosis. In contrast, the other two conidial cells remained viable and contained mainly non-oxidized lipids (**Figure 2A**). As opposed to the wild type, conidial cells of the *ict1*Δ and *ccc2*Δ primarily accumulated non-oxidized lipids at the same time points (**Figure 2A**), indicating that such mutants are unable to trigger lipid peroxidation to lethal levels and thus are defective in conidial Ferroptosis and remain viable. Since *ict1*Δ and *ccc2*Δ responded differentially to iron or copper treatment in restoring cell death, and *ict1*Δ showed stronger Ferroptosis defects, we focused on *ict1*Δ for subsequent experiments.

To ascertain whether the Ferroptosis defect observed in *ict1*Δ conidia is indeed caused by iron deficiency therein, iron levels in wild-type and *ict1*Δ conidia were assessed using Calcein-AM fluorescence, which quenches upon binding to iron (Shen et al., 2020). As has been reported, iron accumulates as a gradient in the three conidial cells of wild type at 4 hpi, with the proximal conidial cell linked to the appressorium containing the lowest level of labile iron, which subsequently also increases to a higher level at 6 hpi during pathogenic development (**Figure 2B**). Such labile iron, however, was significantly low in the *ict1*Δ conidial cells as compared to the wild type at both the time points (**Figure 2B**), indicating an overall iron deficiency in the *ict1*Δ mutant. Taken together, the data presented here confirms the role of Ict1 in mediating the iron- and lipid peroxidation-dependent Ferroptosis in *Magnaporthe.*

### 3.3 Ict1 is necessary for the Ferroptosis-associated function(s) and stability of mitochondria

**Figure 3:**
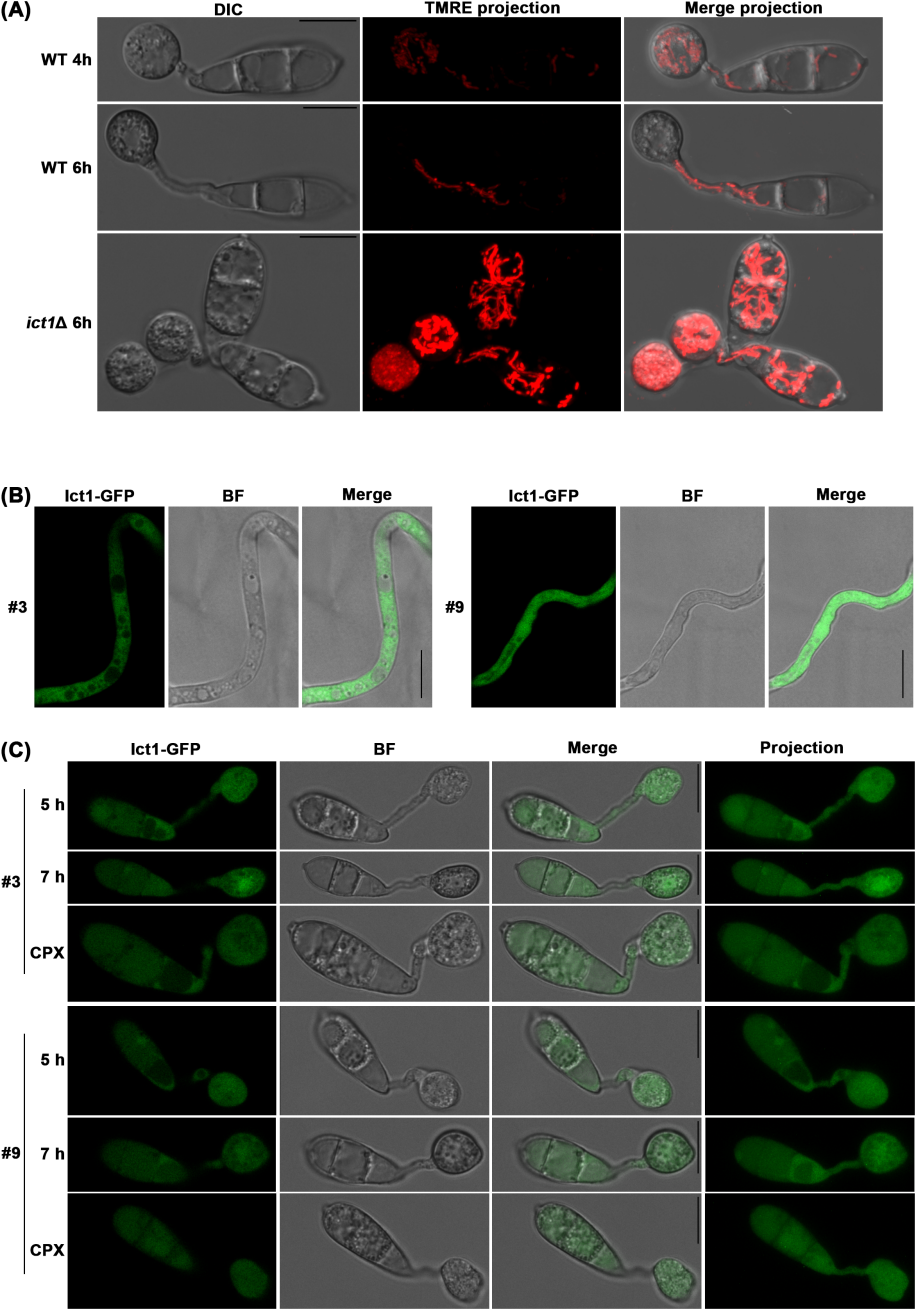
Ict1 is cytosolic and mediates iron-related activities therein during Ferroptosis. **(A)** Mitochondrial stability and membrane potential are modulated by the iron/copper chaperone Ict1 in rice blast. Mitochondrial membrane potential (TMRE stained) is significantly increased in *ict1*Δ conidia that fail to undergo Ferroptosis and retain a stabilized network of mitochondria in direct contrast to the wild-type *M. oryzae* (WT) which sequentially initiates mitophagy in the dying conidial cells and shows reduced membrane potential in each instance. Full view projections merging bright field and TMRE images are presented for clarity. **(B, C)** Ict1-GFP predominantly localizes to the cytoplasm during vegetative growth and early stages of pathogenic development. Confocal micrographs depicting subcellular localization of Ict1-GFP as single plane images or projections within the cell death window in wild-type *M. oryzae* strain in the presence or absence of the iron chelator CPX (5 µM) from 5 to 7 hpi. Chelation of iron fails to affect the cytoplasmic localization pattern of Ict1-GFP. BF refers to bright field image to outline the cell types. Results shown for 2 independent isolates (#3 and 9) of the *ICT1-GFP* strain. Scale bars equal 10 µm.

We previously showed that the mitochondrial membrane potential increases when cellular iron is chelated by CPX (Shen et al., 2024). Since *ict1*Δ suffers from iron shortage, mitochondria in such iron-starved mutant were also examined for differences in membrane potential using the fluorescent dye TMRE. Indeed, a dramatic increase in mitochondrial membrane potential was observed in *ict1*Δ as compared to the wild type, and such presumably active mitochondria were also more abundant in *ict1*Δ than in the wild-type *M. oryzae* (**Figure 3A**). Thus, an increase in mitochondrial membrane potential seems to be constantly associated with iron deficiency. However, whether it represents an alternative mechanism in alleviating iron deficiency when Ict1 is non-functional remains to be addressed. To better understand Ict1-based modulation of Ferroptosis, the subcellular localization of Ict1 was assessed using an in-locus tagged strain expressing Ict1-GFP fusion under its native regulation (**Supplementary Figure 1**). Like the yeast counterpart, Ict1-GFP showed a typical cytosolic localization during vegetative growth in *Magnaporthe* (**Figure 3B**). Such cytosolic localization was also predominant during the pathogenic development, although a weak vacuolar accumulation of Ict1-GFP was evident in some of the conidial cells (**Figure 3C**). Lastly, the Ict1-GFP localization pattern in the cytosol was not affected by iron chelation exerted by exogenous CPX in rice blast (**Figure 3C**). It was worth noting that the Ict1-GFP was distributed uniformly and equally in the 3 conidial cells (**Figure 3C**) during cell death, thus excluding Ict1 as the likely contributor to the specific gradient-based iron accumulation observed in the blast fungus. Taken together, our results helped establish Ict1 as an important cytosolic mediator of cellular iron availability or distribution, and as an essential regulator of the developmental cell death process and fungal pathogenesis. In conclusion, Ferroptosis is regulated by the heavy-metal-associated cytosolic cation chaperone function via cellular iron and redox homeostasis in the rice blast fungus.

## 4 Discussion

Rice blast fungus undergoes iron-dependent cell death, Ferroptosis, in the conidium as an essential step in its pathogenic development prior to plant invasion. Here, we present studies of two HMA-domain containing proteins, Ict1 and Ccc2, that regulate Ferroptosis through cellular iron homeostasis and redox balance. Ortholog of Ict1 in yeast, Atx1, functions as a cytosolic copper chaperone and delivers copper from the cell-surface copper transporter Ctr1 to the copper transporter Ccc2 which finally inserts it into the multicopper oxidase Fet3, which is involved in high-affinity iron uptake. Ccc2, however, can also get copper through endocytosis in an Atx1 independent manner (Lin et al., 1997). Interestingly, unlike iron, exogenous copper treatment does not seem to play an important role in Ict1 function during Ferroptosis in *Magnaporthe*. However, similar to the yeast counterpart, Ict1 displays a typical cytosolic localization but regulates cellular iron homeostasis instead, probably through iron release from internal sources, and thus regulates Ferroptosis through timely iron availability in *Magnaporthe*. Ferroptosis related defect in *ict1*Δ, particularly the decrease in the levels of lipid peroxidation, was not as strong as in *ccc2*Δ, implying that Ccc2 in *M. oryzae*, is able to obtain copper through an Ict1- independent mechanism. Supporting such a hypothesis, Ccc2 in *M. oryzae* contains more HMA domains as compared to Ict1. It remains unclear whether additional Ccc2-independent copper transport mechanisms exist and account for such cell death variation observed in *ccc2*Δ (**Figures 1C, D**).

Like Atx1 and Ccc2, the downstream Fet3 and the high affinity iron permease Ftr1 are also conserved in *M. oryzae*, but the upstream copper transporter Ctr1 is not, which raises the complexity of the source of copper in *Magnaporthe*. Furthermore, unlike yeast Atx1, iron deficiency caused by loss of Ict1 cannot be rescued/suppressed by copper treatment. Therefore, whether Ict1 also acts as a copper chaperone in the cytosol, or its function has been adapted or modified in *M. oryzae* for direct iron binding and transport remains an open question for future research. On the other hand, Ict1 function, in terms of transport and/or release of iron from internal sources, probably reaches the maximal capacity once it is switched on. Supporting such a hypothesis, the abundance and subcellular localization of Ict1-GFP showed no response to CPX-based iron chelation (**Figure 3C**). In addition to the cytosol, a weak vacuolar distribution of Ict1-GFP was also observed in some conidial cells, particularly during pathogenic development. Since fungal mycelia can take up iron from the culture media during vegetative growth, such a different Ict1 localization pattern may reflect iron acquisition from diverse intrinsic sources during the infection cycle. However, it is also plausible that Ict1-GFP is targeted to the vacuole for degradation. It is worth noting that Ict1-GFP distributed equally in the 3 conidial cells (**Figure 3C**), thus excluding Ict1 as the likely contributor to the specific gradient-based sequential iron accumulation observed in the blast fungus (**Figure 2B**).

Timely clearance of dysfunctional or excess mitochondria via mitophagy is reported to be required for Ferroptosis, and loss of mitophagy leads to iron starvation and also a decrease in mitochondrial membrane potential (Shen et al., 2024). Here, in the iron deficient *ict1*Δ, abundant stable mitochondria with high levels of mitochondrial membrane potential were observed. It remains unclear whether such mitochondrial response is specific to iron deficiency and represents an alternative mechanism to fix the iron shortage within the cells or whether the lack of iron (upon loss of Ict1) also influences the organellar redox homeostasis therein. Furthermore, the specificity and dynamics of the cation binding (iron vs copper) in Ict1 and its role in regulating Ferroptosis needs further enquiry. Ferroptosis is known to be essential for pathogenesis in the rice blast fungus (Shen et al., 2020). Our preliminary data (**Supplementary Figure S4**) corroborate the prior study on the essential function of Ict1 and Ccc2 in *M. oryzae* pathogenesis in barley (Harata et al., 2020) and further suggest a minor role during vegetative growth and asexual development (**Supplementary Figure S5**). Future experiments will help address the requirement of such iron-copper transport or chaperone functions in *Magnaporthe* pathogenesis towards rice, and in deciphering the cation specificity of the HMA motif and/or the crosstalk, if any, between Ict1 and Ccc2 activity in the blast pathosystem. Overall, a novel Ferroptosis regulator, Ict1, that potentially links iron and copper homeostasis with mitochondrial functions was identified and characterized in *M. oryzae*. Knowledge gained here may contribute to an optimized cessation of Ferroptosis as an innovative strategy for blast disease control and crop protection.

## Data Availability

The original contributions presented in the study are included in the article/**Supplementary Material**. Further inquiries can be directed to the corresponding authors.
