# Supplementary material for: The HMA-domain protein Ict1 is required for Ferroptosis in the rice blast fungus
Source: Front Plant Sci. 2025 Jun 5;16:1576086. doi: 10.3389/fpls.2025.1576086 (PMC12176895; doi:10.3389/fpls.2025.1576086)
Supplement: Supplementary file 1 [file DataSheet1.docx]

**Shen et al., Supplementary Material**

**
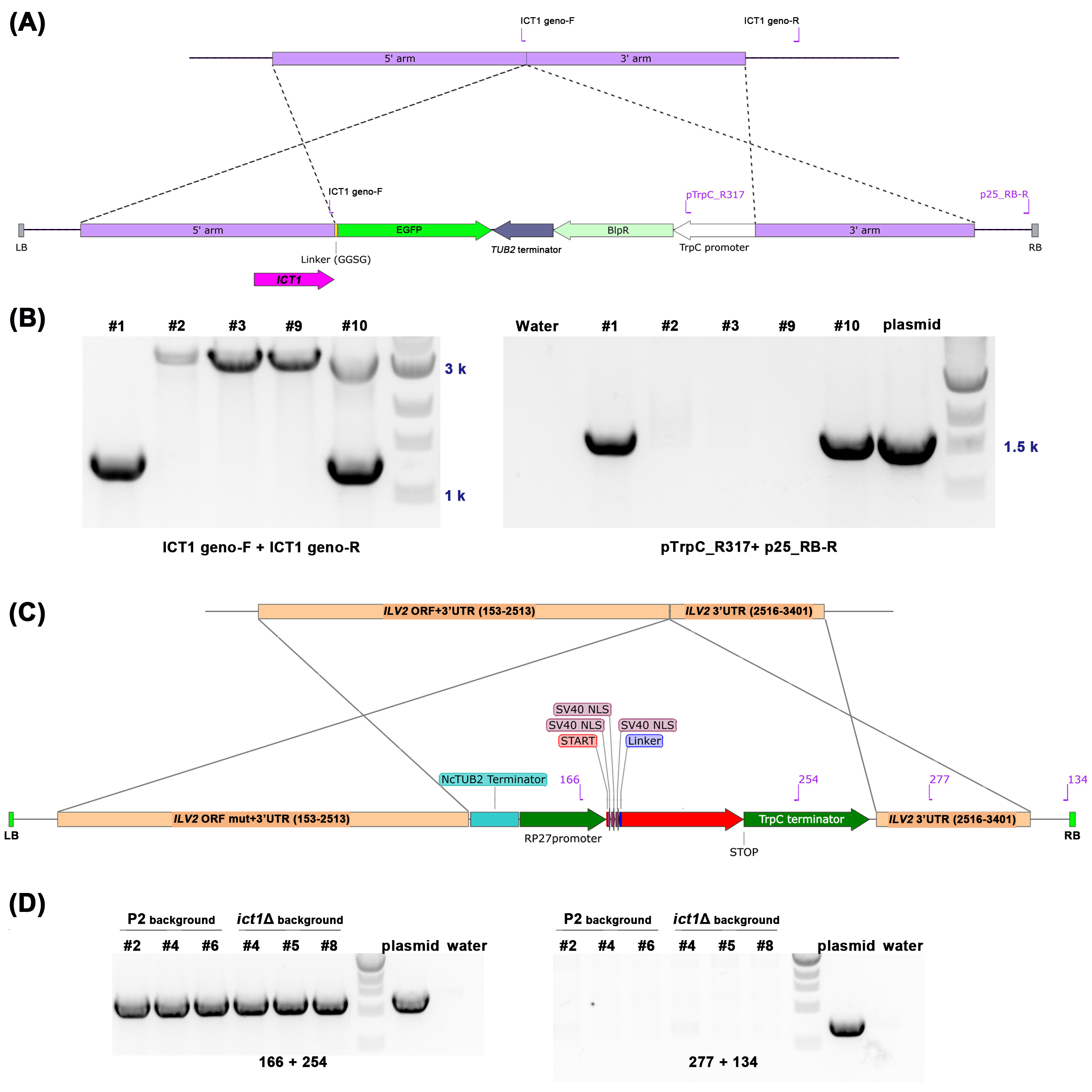
Supplementary Figure 1**

**Supplementary Figure 1.** Generation and verification of in-locus tagged *ICT1-GFP* strain. **(A)** Schematic descriptor for the plasmid vector used for homologous recombination-based GFP tagging of Ict1. **(B)** PCR-based strain verification and confirmation using the indicated primers. ICT1 geno-F and ICT1 geno-R are the set of primers used to confirm correct insertion of GFP and the Basta resistance cassette at the *ICT1* locus, while pTrpC_R317 and p25_RB-R are primers used to examine and avoid random insertion event(s), if any. **(C)** Schematic depiction of the strategy for generating the nucleus-tagged (3xNuclear Localization Signal fused to mCherry) strain of *M. oryzae* wild type or *ict1*Δ mutant*.* **(D)** Verification and confirmation of the *3xNLS-mCherry* strain using standard PCR-based amplification with the indicated primers. PCR primer set 166 and 254 was used to confirm the correct insertion of 3xNLS-mCherry cassette at the *ILV2* locus, while 277 and 134 are primers used to examine and avoid random insertion event(s), if any.

**
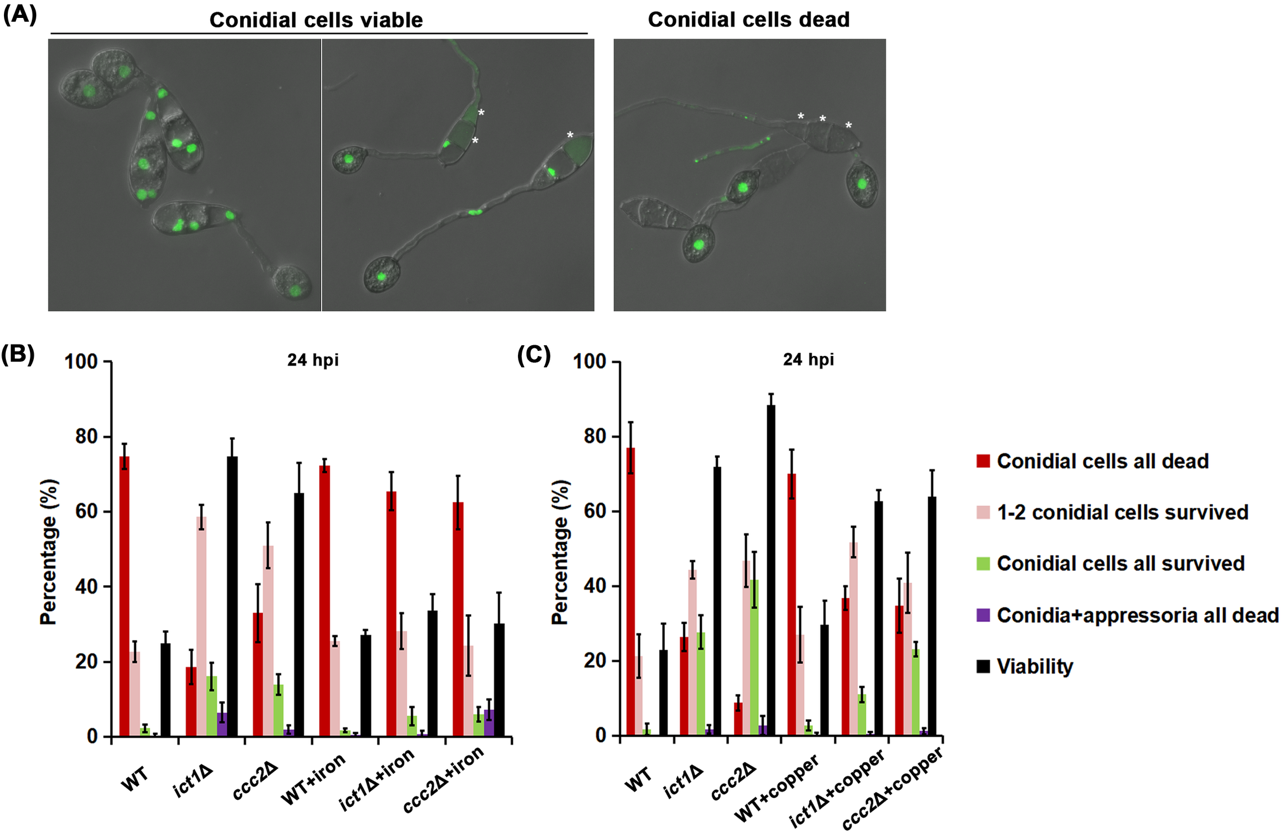
Supplementary Figure 2**

**Supplementary Figure 2.** **(A)** Method for quantification of ferroptosis-dependent cell death in conidia using nuclear degeneration as a proxy as schematized in Figure 1B. Conidia from the nucleus-tagged *Histone H1-GFP* strain of *M. oryzae* showing cell death (asterisk; nucleus completely degenerated with the GFP signal either vacuolar or degraded) occurring sequentially in the three cells of the conidium starting with the cell that is most distal to the infection structure or appressorium. At the maturity stage i.e. 24 h post inoculation, all conidial cells undergo such developmental ferroptosis leaving only the appressorium viable with an intact nucleus. **(B)** Loss of Ict1 or Ccc2 leads to marked reduction in Ferroptotic cell death, which can be significantly restored by exogenous iron in the rice blast fungus. Conidial cell viability (green or pink; black) or death (red; magenta) in the wild type (WT), *ict1*Δ or *ccc2*Δ was quantified at 24 hpi in the presence or absence of the indicated amounts of iron/ferric ions. Data presented as mean ± SD (3 technical replicates, n=100 conidia for each time point per strain per replicate). ** (p < 0.01) and * (p < 0.05) indicate significant differences, while n.s. refers to no significant difference detected in comparison to the WT at the corresponding time points. Experiment was repeated thrice. (**C**) Exogenous copper marginally restores cell death in the *ict1*Δ or *ccc2*Δ conidia. Conidial cell viability (green or pink; black) or death (red or magenta) in the wild type (WT), *ict1*Δ or *ccc2*Δ was quantified at 24h in the presence or absence of copper ions. Data presented as mean ± SD (3 technical replicates, n=100 conidia for each time point per strain per replicate). ** (p < 0.01) and * (p < 0.05) indicate significant differences, while n.s. refers to no significant difference detected in comparison to the WT at the corresponding time points. Experiment has been repeated thrice.

**
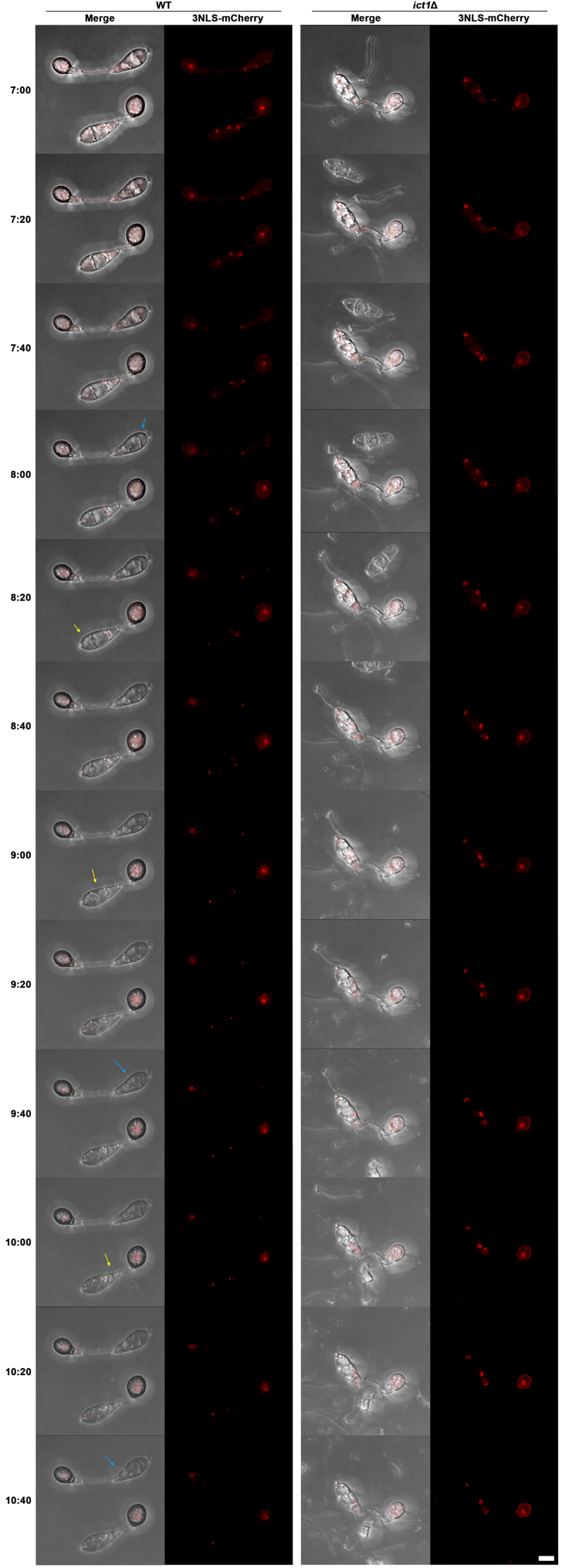
Supplementary Figure 3**

**Supplementary Figure 3.** The *ict1*Δ conidia fail to undergo Ferroptosis cell death. Montage from time-lapse confocal imaging of the 3xNLS-mCherry expressing wild-type or *ict1*Δ *M. oryzae* strain showing the dynamics and temporal control of sequential conidial cell death (arrows) that starts first in the terminal cell (distal from the appressorium), and spreads subsequently to the middle and the proximal cell. Loss of Ict1 leads to a complete lack of such developmental cell death and renders the conidial cells viable. Merge refers to integrated overlap of the corresponding bright field and mCherry confocal micrographs. Data shown are representative of the analysis of at least 10-20 conidia in each strain across 2 independent experiments that showed the same pattern or sequence of conidial cell death in wild-type or cell viability in the *ict1*Δ mutant, respectively. Indicated time is hours post inoculation (hpi). Bar, 10 μm.

**Supplementary Figure 4**

**
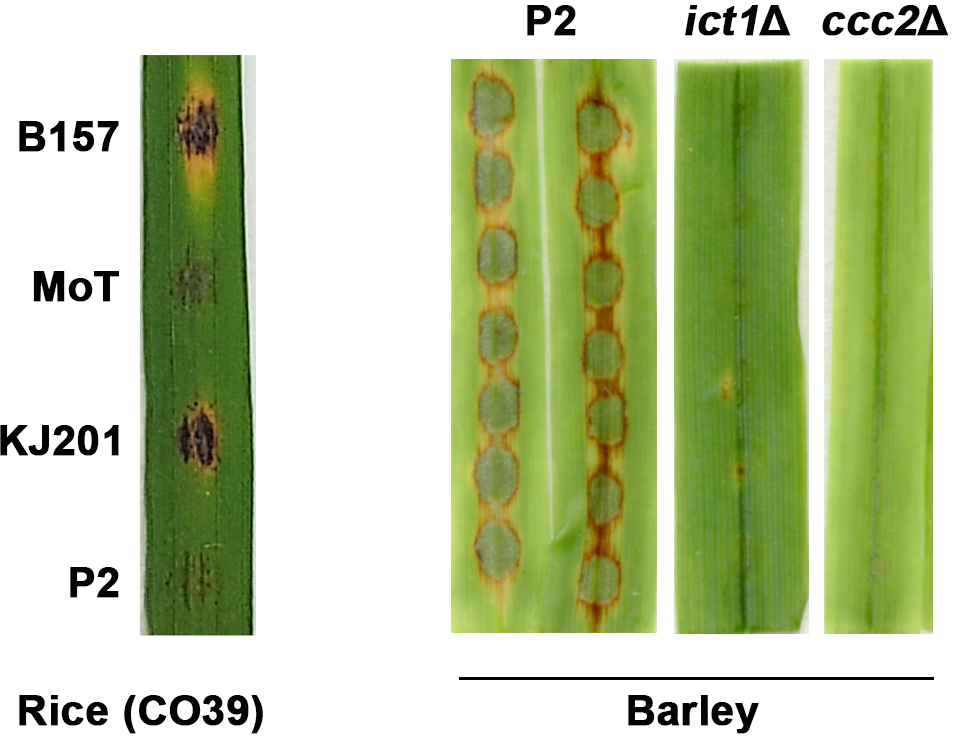
**

**Supplementary Figure 4.** Loss of Ict1 or Ccc2 renders *M. oryzae* incapable of causing blast disease in barley. Conidia from the indicated wild-type or the *ict1*Δ or *ccc2*Δ mutant strain of *Magnaporthe* were inoculated on rice or barley leaf explants, and the disease symptoms assessed 7 days post inoculation. Conidia load per droplet was either 5x10^3^ (for rice) or 4x10^3^ (for barley). Inoculated leaf explants were cultured on 1% kinetin agar medium at 24^0^C with 90% relative humidity and 16h:8h light:dark cycle (For more details, please refer to Shen et al., New Phytologist 2020). MoT refers to the wheat blast isolate, which is non-pathogenic on rice plants and served as a negative control. P2 is incompatible with and fails to cause blast disease in rice variety CO39. The *ict1*Δ or *ccc2*Δ mutant is non-pathogenic on barley. Representative images from 3 independent experiments are shown.

**
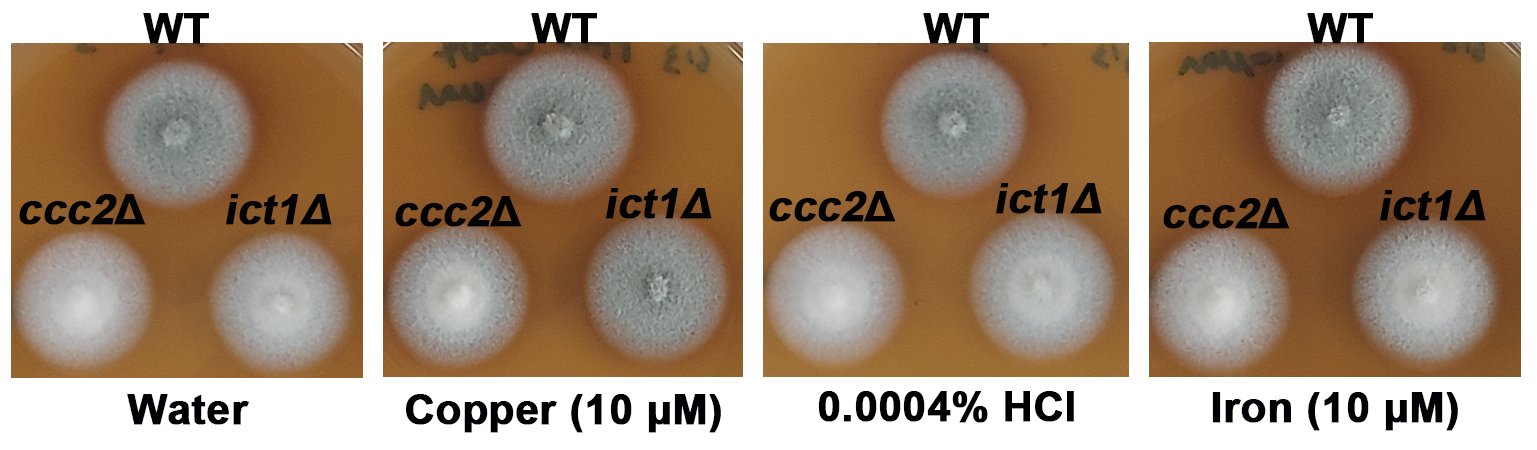
Supplementary Figure 5**

**Supplementary Figure 5.** Exogeneous iron or copper have minimal effect on vegetative growth or colony characteristics in the *ict1*Δ or *ccc2*Δ mutant. Mycelial plugs from the wild type, *ict1*Δ or *ccc2*Δ were cultivated on prune agar medium in the presence or absence of the indicated amount of cation (iron or copper at 10 μM each) and the results documented at 7 dpi. Water or dilute hydrochloric acid served as a solvent/mock control.

**Supplementary Table S1. List of oligonucleotide primers used in this study**

| **Name** | **Sequence (5' to 3')** | **Purpose** |
| --- | --- | --- |
| 5’UTR ICT1-F | 5'-tatggagaaactcgagaattcAGATTGGGAGGAGAGTGACGG -3' | Vector construction |
| 5’UTR ICT1-R | 5'- tgctcacacccgatccgccTGCCGAAGGGACCTCGATgc -3' |  |
| GFP BAR-F | 5'- aGGCGGATCGGGTGTGAGC -3' |  |
| GFP BAR-R | 5'- ggggttttctaGTCGAGGATATTGAAGGAGCACTT -3' |  |
| ICT 3’UTR-F | 5'- atcctcgacTAGAAAACCCCGCTTTTGGG -3' |  |
| ICT 3’UTR-R | 5'- gactctagaactagtggatccTCCTTTCGGGTTGTCAGCTC -3' |  |
| ICT1 geno-F | CAGAGCATCGAGGTCCCTTC | Verify in-locus tagging |
| ICT1 geno-R | ATCGGCAGAAAGTTTGGGGT |  |
| pTrpC_R317 | AATAAAGGGAGGAAGGGCGAACT | Verify no extra or random insertion(s) |
| p25_RB-R | ACTGAAGGCGGGAAACGACAATCTGA |  |
